# Pervasive Cryptic Epistasis in Molecular Evolution

**DOI:** 10.1371/journal.pgen.1001162

**Published:** 2010-10-21

**Authors:** Mark Lunzer, G. Brian Golding, Antony M. Dean

**Affiliations:** 1 BioTechnology Institute, University of Minnesota, St. Paul, Minnesota, United States of America; 2 Department of Biology, McMaster University, Hamilton, Ontario, Canada; 3 Department of Ecology, Evolution and Behavior, University of Minnesota, St. Paul, Minnesota, United States of America; 4 Instituto Gulbenkian de Ciência, Oeiras, Portugal; University of Toronto, Canada

## Abstract

The functional effects of most amino acid replacements accumulated during molecular evolution are unknown, because most are not observed naturally and the possible combinations are too numerous. We created 168 single mutations in wild-type *Escherichia coli* isopropymalate dehydrogenase (IMDH) that match the differences found in wild-type *Pseudomonas aeruginosa* IMDH. 104 mutant enzymes performed similarly to *E. coli* wild-type IMDH, one was functionally enhanced, and 63 were functionally compromised. The transition from *E. coli* IMDH, or an ancestral form, to the functional wild-type *P. aeruginosa* IMDH requires extensive epistasis to ameliorate the combined effects of the deleterious mutations. This result stands in marked contrast with a basic assumption of molecular phylogenetics, that sites in sequences evolve independently of each other. Residues that affect function are scattered haphazardly throughout the IMDH structure. We screened for compensatory mutations at three sites, all of which lie near the active site and all of which are among the least active mutants. No compensatory mutations were found at two sites indicating that a single site may engage in compound epistatic interactions. One complete and three partial compensatory mutations of the third site are remote and lie in a different domain. This demonstrates that epistatic interactions can occur between distant (>20Å) sites. Phylogenetic analysis shows that incompatible mutations were fixed in different lineages.

## Author Summary

Many bioinformatics and functional genomics predictions are derived from evolutionary patterns of amino acid replacement in protein sequence alignments. Most computational methods assume that replacements in one sequence will be tolerated in all related sequences. Here, we evaluate—by direct experiment—the functional impact of amino acid replacements accumulated during the course of evolution. Our initial results show that cryptic interactions among amino acid replacements are common and that most are deleterious. This result has implications not only for the evolution of function, recombination, sex, dominance, robustness, disease, and even speciation, but also for practical applications—in conservation biology (e.g. to decide which organisms to preserve) and in vaccine design (e.g. using consensus or reconstructed ancestral sequences). Analyzing one interaction in detail, we find that compensatory mutations need not lie in close proximity to the original mutation as generally supposed. This result suggests that unsuspected structure–function relationships can be revealed by analyzing patterns of site-to-site interactions among amino acid replacements in evolution.

## Introduction

In a half century of molecular phylogenetics there never has been a systematic investigation of the functional and fitness effects of amino acid replacements in evolution. Experimental studies focus on those few mutations that change protein function [1]. Of the remaining thousands of replacements nothing is said – they may or may not be of functional consequence. Sequence analyses use statistical approaches to explore modes of evolution [2], [3]. Rarely does fitting alternative evolutionary models to observed data disallow alternative explanations. For example, to some [4]–[6] an elevated ratio of amino acid replacements to silent substitutions between species (*d_n_*/*d_s_*) suggests evidence for the action of positive selection. To others [7] it suggests relaxed selection against slightly deleterious amino acid replacements during population bottlenecks. Both interpretations are viable.

Non-additive interactions among mutations (epistasis) are critical to protein structure and function [1], [8] and consequently to speciation [9], the evolution of sex [10], recombination [11], dominance [12], robustness [13] and human disease [14]. Non-additive interactions force sites to functionally co-vary during evolution [15], [16]. Computational methods that ignore phylogenetic structure [17]–[29] fail to distinguish between co-variation arising from functional causes and co-variation arising though shared common ancestry. The latter, an ineluctable product of shared history, is reflected in the bifurcating hierarchy of a phylogenetic tree (a tree must collapse to a star burst if no sites co-vary). Computational methods that account for phylogenetic structure [30]–[38] have identified sites likely to functionally co-evolve [32], [36]. The relative scarcity of such sites accords with the observation that most amino acid replacements occur at the surfaces of proteins where solvent exposed side chains are less likely to interact [39], [40]. On the other hand it may simply reflect a lack of statistical power in many, though not all [32], of computational methods used. An alternative approach identifies pathogenic missense mutations in one species that have no obvious detrimental effect in a related species [41], [42]. This approach does not detect deleterious mutations of minor phenotypic effect.

Computationally derived predictions need empirical verification [1]. Gloor et al. [43] used site directed mutagenesis to confirm the epistasis predicted between co-evolving residues in yeast phosphoglycerate kinase. Experiments with yeast iso-2-cytochrome *c* [44] also identified epistatic interactions between sites. However, two other studies, one with game bird lysozymes [45], [46] and one with vertebrate p53 domains [47], failed to find any evidence of epistasis. Several other site directed mutagenesis studies identified epistatic interactions among positively selected replacements in TEM-1 β-lacatamase [48], vertebrate steroid receptors [49] and visual pigments [50] and in coral red fluorescent proteins [51]. In no case, however, have experiments been designed to explore the prevalence of epistasis in molecular evolution in general.

Here, we explore the prevalence of epistasis in molecular evolution from the distribution of functional effects caused by individual mutations introduced to one sequence from a homologue in another species. We studied the *leuB* encoded β-isopropylmalate dehydrogenase (IMDH) because: 1) the enzyme has a conserved well defined role in leucine biosynthesis [52], [53]; 2) high resolution x-ray crystallography of divergent IMDHs (<35% identical) reveals a conserved protein fold [54]–[57]; sequence alignments show that divergent IMDHs rarely differ by more than a few insertions and deletions; and 4) the relationship between enzyme performance (*k_cat_/K_m.NAD_*) and fitness has been determined using *Escherichia coli* as a model system [58]. The IMDHs from two mesophyles, *E. coli* and *P. aeruginosa*, differ at 168 of 365 sites including six small indels (Figure S1) located in flexible loops external to the core structure. Conserved in fold and function, *E. coli* and *P. aeruginosa* IMDHs provide excellent material with which to investigate protein evolution arising through sequence divergence in the absence of major changes in structure and function.

## Results/Discussion

### Functional Effects of Single Amino Acid Replacements

**Figure 1:**
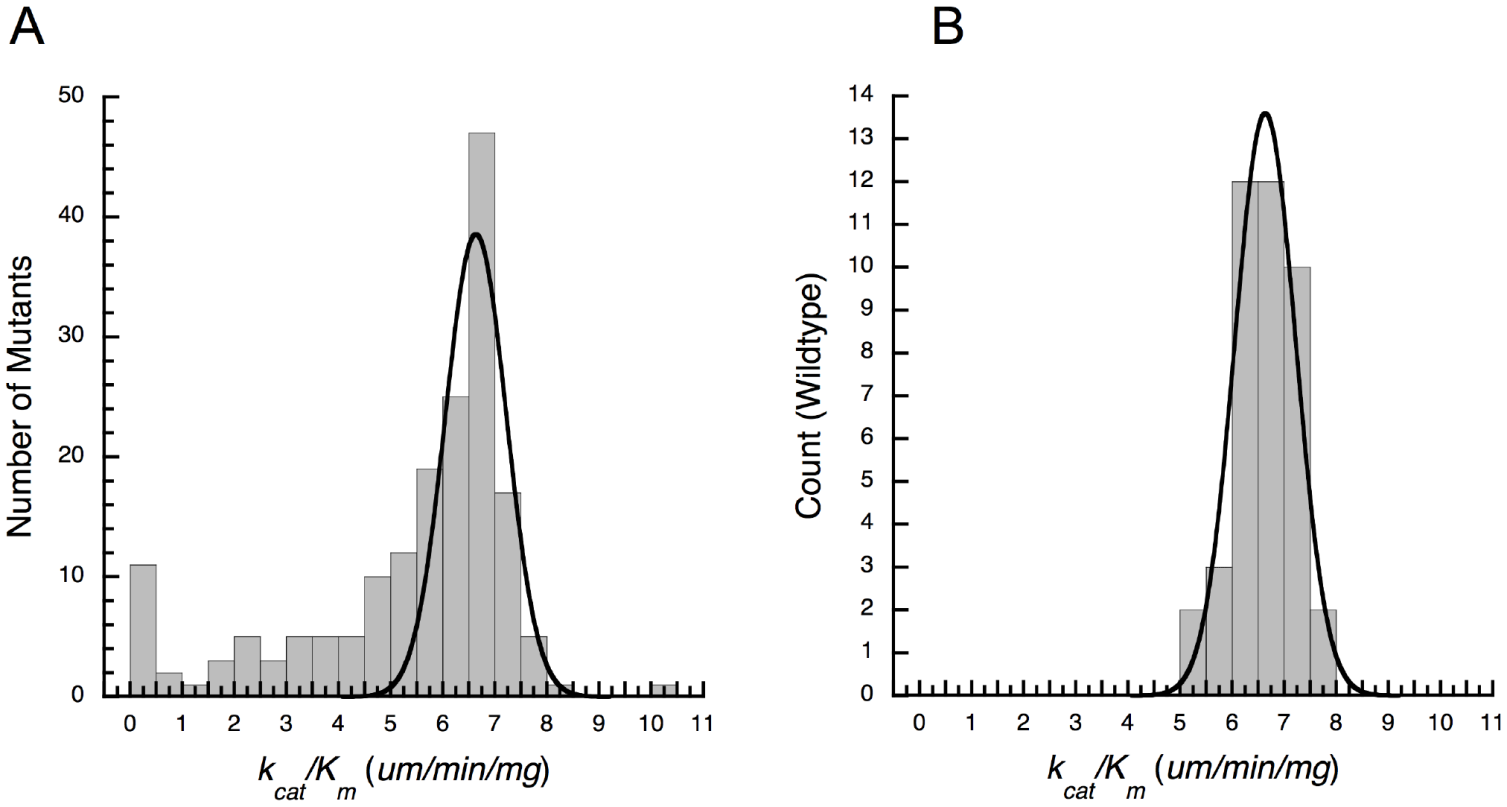
Cryptic epistasis in IMDH. (A) Distribution of functional effects produced upon moving single amino acids and indels from *P. aeruginosa* IMDH into *E. coli* IMDH. The shoulder on the left indicates the presence of extensive epistasis. (B) Error distribution obtained by repeatedly assaying wild-type *E. coli* IMDH. Gaussian distributions fitted as described in the text.

We constructed 168 site directed mutants of *E. coli leuB* (each with a single mutation from *P. aeruginosa leuB*) and then expressed and purified each enzyme and determined its kinetic parameters. The resulting distribution of enzyme performances (*k_cat_/K_m.NAD_*) is strongly skewed to the left and only a single outlier with increased performance lies on the right (Figure 1A). The error distribution, obtained by repeatedly assaying wild-type *E. coli* IMDH, is Gaussian, 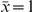, 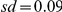 (Figure 1B). This same distribution is expected of amino acid replacements that do not affect function. The 52 mutants with relative performances above *E. coli* wild-type form a half-Gaussian distribution, 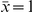, 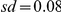, similar to the error distribution. This suggests 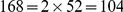 mutations have no detectable effect on enzyme performance, 63 reduce it, and one increases it. Pair-wise *t*-tests (for unequal replication and unequal variances [59]) combine with a 5% false discovery rate [60] to identify 61 mutants of changed performance: 56 have decreased performance and 5 have increased performance, with only the single outlier having performance increased by more than 15%.

The assumption that mutations act independently, and hence additively, leads to a predicted performance for *P. aeruginosa* IMDH that is clearly wrong. The sum of the individual mutational effects and the *E. coli k_cat_/K_m.NAD_* is negative: 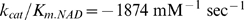. This is a physical impossibility. The assumption that mutations act multiplicatively is also wrong. In simple transition state theory 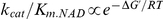, where *ΔG*′ is the difference in free energy between the ground state and the transition state, *R* is the gas constant and *T* is °Kelvin [61]. The difference in free energy between the transition states of the mutant and wild-type enzymes is 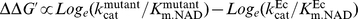. With five mutants completely inactive the sum of the *ΔΔG*′ and 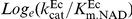 is minus infinity and the predicted performance is 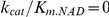. In fact *P. aeruginosa* IMDH has a 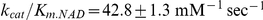, 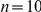, slightly lower than that of *E. coli* IMDH which has a 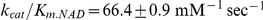, 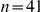. The inescapable conclusion is that amino acid replacements at many sites interact. IMDH evolution is characterized by rampant epistasis that remains cryptic until revealed by experiment.

**Figure 2:**
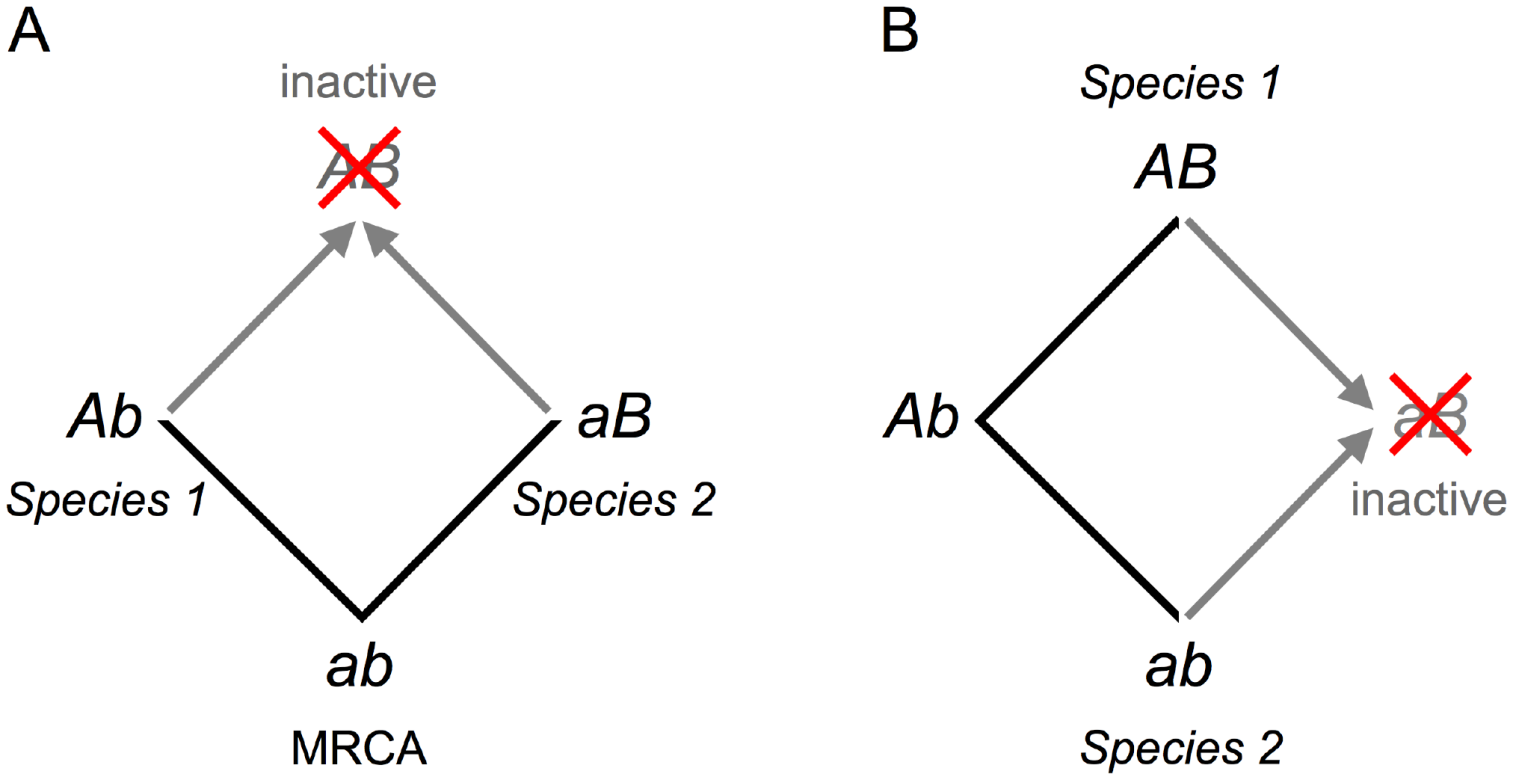
The evolution of cryptic epistasis. (A) Two lineages diverge from the most recent common ancestor (MRCA), genotype *ab*. Each fixes a mutation to produce genotypes *Ab* and *aB*. The presence of either mutation prevents the second becoming fixed because the double mutant, genotype *AB*, is deleterious. Cryptic epistasis is revealed upon moving mutation *A* from species 1 into species 2 or moving mutation *B* from species 2 into species 1 (gray arrows). Moving mutations *a* or *b* to form genotype *ab* restores the ancestral functional state. (B) Cryptic epistasis can also occur when both mutations arise in the same lineage leading to species 1. In this case cryptic epistasis is revealed upon moving mutation *a* into species 1 or mutation *B* into species 2 (gray arrows). Moving mutations *A* or *b* to form genotype *Ab* regenerates a functional evolutionary intermediate.

That only 64 of the 168 sites affect function certainly underestimates the number that interact epistatically. To understand this, consider two cases in which two sites are involved in a simple pair-wise interaction (Figure 2). In each case, only one of the two sites reduces function when an amino acid in one species is mutated to that found in another. Mutating the second site restores the ancestral functional state. Mutations at both sites may reduce function if three or more amino acid replacements arose during the course of evolution (Figure S2A) – although this is not guaranteed. In a simple network of pair-wise interactions only two of three sites might be identified (Figure S2B). We expect some of the remaining 104 sites to engage in epistatic interactions.

Zuckerkandl [15] first proposed that amino acid replacements at one site in a protein might influence the acceptability of amino acid replacements at other sites. Fitch and Markowitz [16] suggested that as species diverge from a common ancestor their sets of variable sites also diverge to explore different regions of sequence space. Mutating a currently invariant site in one species by introducing an amino acid from a homologous protein in another species risks producing a loss-of-function mutant. The many functionally compromised mutants in this study amply confirm the insight of these early pioneers.

### Location of Deleterious Amino Acid Replacements

Mutations affecting function (<80% wild-type performance) are scattered throughout the IMDH structure (Figure S3). Solvent accessibility, distance to the catalytic center (Asp 251), secondary structure and rate of amino acid replacement per site do not correlate significantly with performance for the mutants analyzed here. Only one mutation, F73L, likely affects catalysis by contacting a substrate directly (Figure S4).

### Identifying Compensatory Mutations

We screened for compensatory mutations of F73L, A94D and A284C, all of which lie near the active site and all of which are among the least active mutants. We combined each deleterious mutation with each of the 167 remaining mutations, expressed and purified each double mutant, and assayed their activities. Four mutations compensate the F73L mutation (Table 1). F120A, identified in the original screen because it produces a 50% increase in wild-type performance, now produces a 6-fold increase in performance so that the F73L,F120A double mutant, while not as active as the *E. coli* wild-type enzyme, is as active as the *P. aeruginosa* enzyme. Three other compensatory mutants, F132L C136I and I179V, do not individually affect wild-type performance. Performance is completely restored to *E. coli* wild-type levels in the F73L,I179V double mutant. Performance is partly restored in the F73L,F132L and F73L,C136I double mutants. No mutations were found to restore function to A94D and A284C.

**Table 1:** Performance of mutants towards NAD.

	Performance* *k_cat_/K_m.NAD_* (mM^−1^sec^−1^)	
	Mutant	
Enzyme	F73	F73L
*E. coli* wt	166	7
F120A	102	44
F132L	182	25
C136I	172	39
I179V	161	66
*P. aeruginosa* wt		42

*All standard errors <10% of the estimates.

Our results are compatible with several types of interactions. Using *E. coli* IMDH performance as a standard suggests a simple pair-wise interaction between L73 and I179 because only L73F and I179V are fully compensatory. Using the lower *P. aeruginosa* IMDH performance as a standard suggests a high-order interaction with function compromised only when residues L73, F120, C136, I179 are combined. Replacing any one amino acid, L73F, F120A, C136I, or I179V destroys the 4-way interaction to restore full performance. That no mutation restores function to A94D and A284C demonstrates several compensatory mutations are essential; at least two sites (*A* and *B*) must each interact with each original mutation (*X*) to form a simple chain (*A-X-B*).

**Figure 3:**
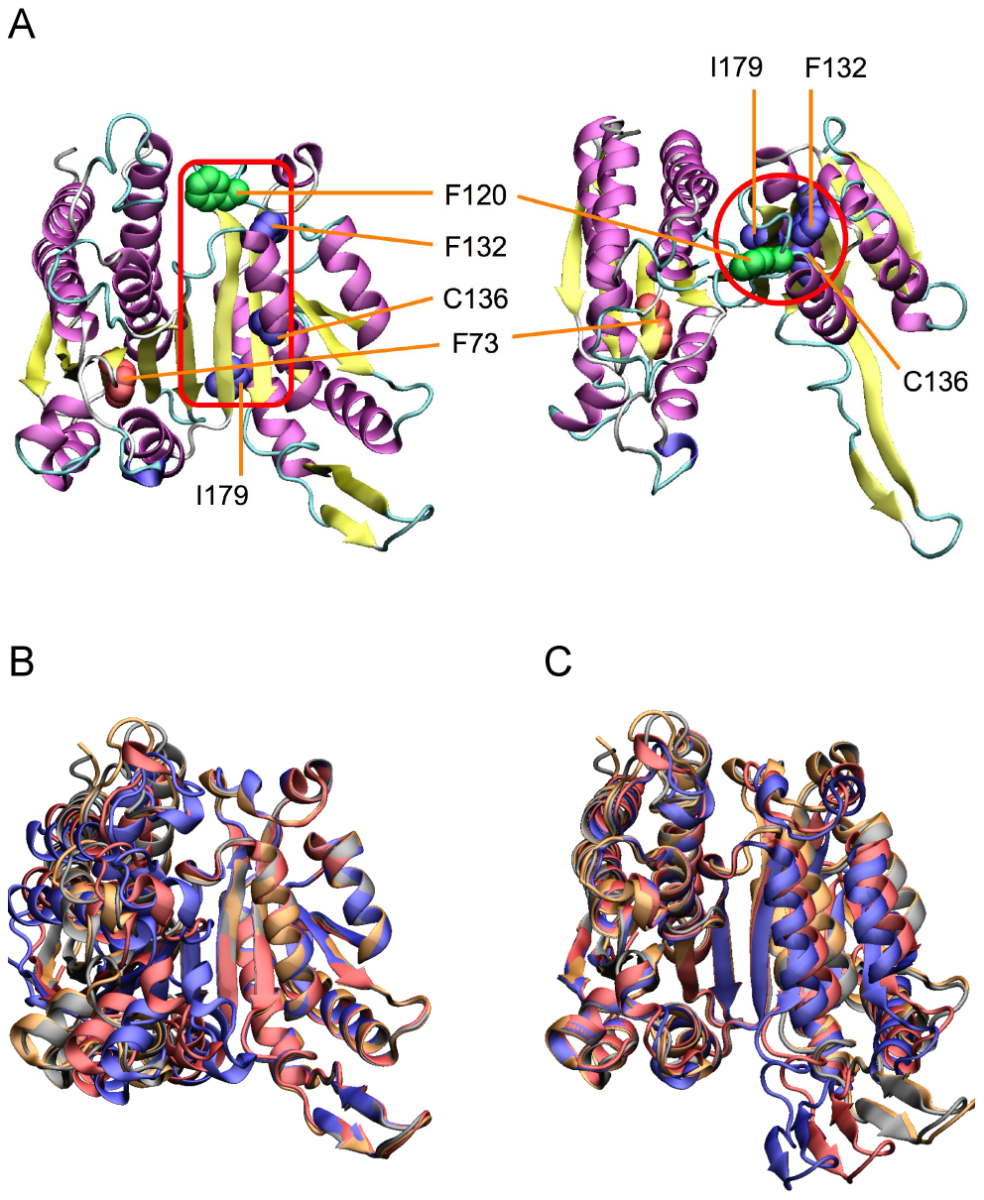
Locations of compensatory mutations for F73L. Compensatory mutations for the deleterious effects of F73L in the large domain are located in the small domain close to the hinge in the b-sheet. (A) Two views of the locations of the compensatory mutations for F73L in *E. coli* IMDH (space filling: brown for F73L, green for F120A and blue for F132L, C136I and I179V). (B) The two domains of IMDH are built on the β-sheet and move as rigid bodies. Superimposing the small right-hand domains of *E. coli* and *S. typhimurium* IMDHs reveals rigid body movements of the large domains on the left. (C) Superimposing the large left-hand domains reveals rigid body movements of the small domains on the right. The hinge region lies in the β-sheet between the two domains. Structures from *E. coli* (pdb 1CM7) and *S. typhimurium* IMDHs (pdb 1CNZ).

Whereas previous experimental studies [41]–[45] assumed interacting residues would be in close physical contact, all compensatory mutations for F73L in the large domain lie more than 20 Å distant in the small domain, close to a hinge in the b-sheet on which the two domains swivel (Figure 3). This suggests a common mode of action, possibly related to repositioning the F73L-shifted nicotinamide ring for catalysis. Our experimental strategy, of moving replacements from one homologue into another and screening for compensatory mutations, is useful in that it provides a general means to identify interacting sites regardless of the mechanisms involved.

### Predicted Fitness Effects

**Figure 4:**
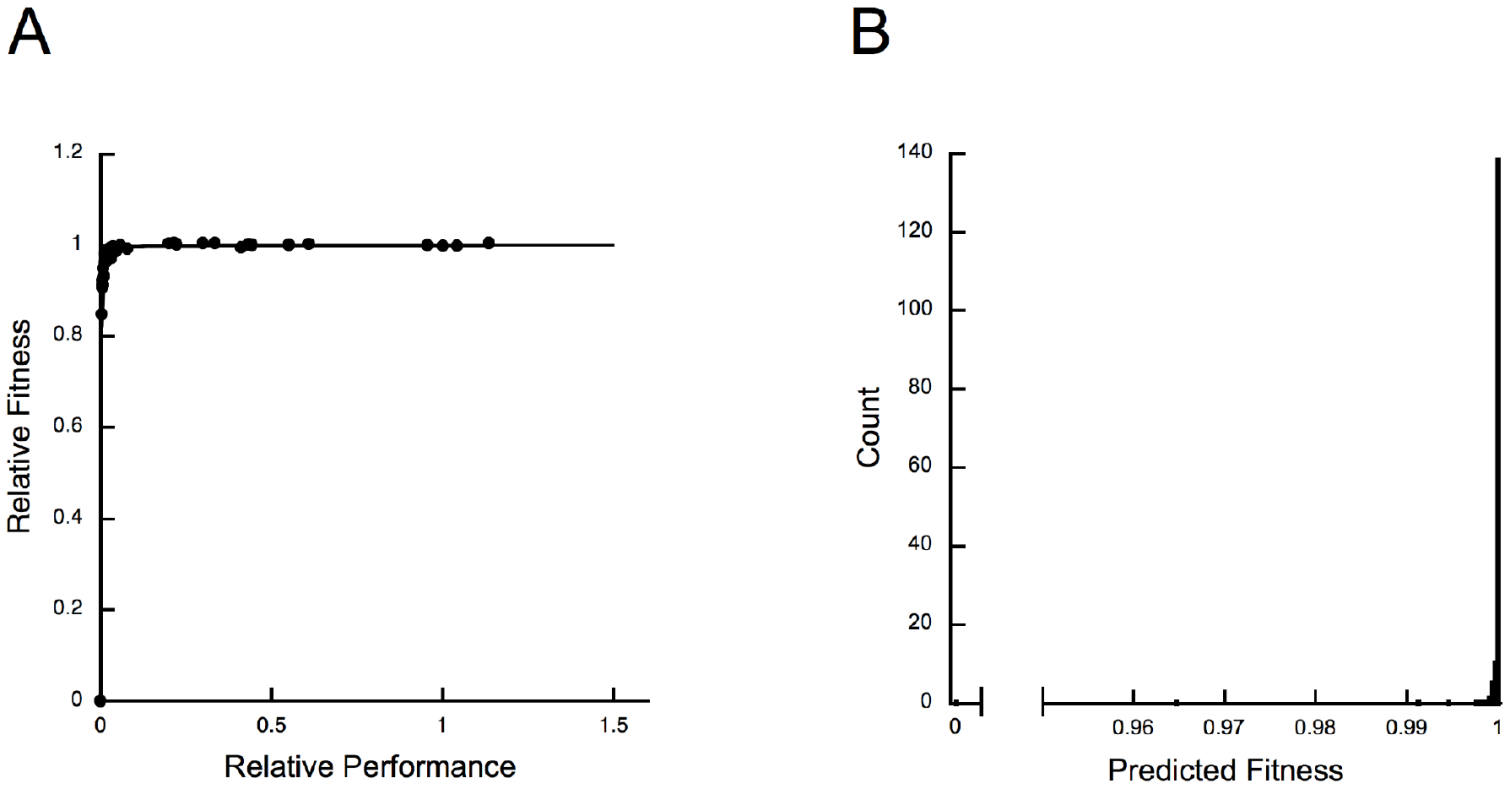
Large changes in IMDH performance cause small changes in fitness. (A) Relationship between fitness and performance, which conforms to a dominance curve [12], shows that *E. coli* wild-type IMDH lies far into a fitness plateau [58], [62]. (B) As a consequence, even large reductions in enzyme activity are predicted to have very small fitness effects. The predicted fitnesses were calculated using a hyperbola, *y* = 1.0005*x*/(0.00032+*x*), fitted to the data in (A).

The predicted fitness effects of most mutations are tiny. Previous work [56] established that wild-type *E. coli* IMDH lies on a fitness plateau (Figure 4A). In this limit of adaptation [62], increases in performance do not improve fitness and even large reductions in IMDH performance produce small fitness effects (Figure 4B). Indeed, 65% of the selection coefficients are predicted to be less than 10^−5^/generation. Selection during starvation growth with glucose as the sole limiting resource is far greater than in nature where leucine is both widely available and abundant [63]. Many mutations, including the one with increased performance, are likely selectively neutral, or very nearly so.

### Two Models of Neutral Evolution

The cryptic epistasis we revealed is consistent with two modes of neutral evolution: the covarion process [64] and the nearly neutral process [65]. In the covarion process, neutral and/or beneficial mutations are fixed in different lineages that, when brought together in the same protein, are deleterious (Figures 2, Figure S2). In the nearly neutral process successive slightly deleterious alleles are fixed by random genetic drift (particularly during population bottlenecks) until a compensatory mutation arises that, on restoring full activity, is fixed by positive selection (particularly after a population expands). The concave fitness function for *E. coli* IMDH (Figure S4), typical of dominance curves [12], provides the fitness plateau on which fitness could gradually drift downwards as slightly deleterious mutations sequentially fix before a beneficial compensatory mutation restores full activity.

**Figure 5:**
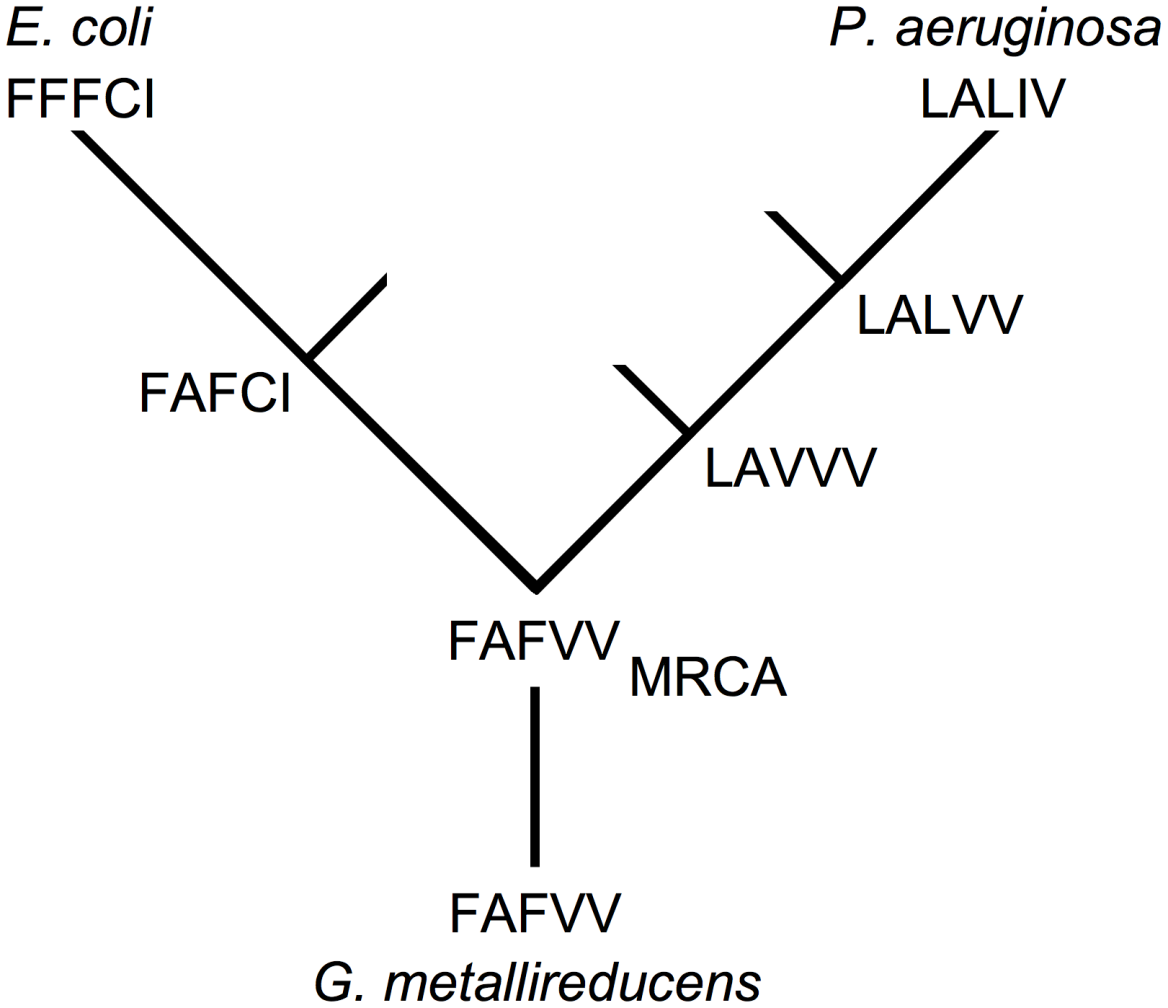
Incompatible mutations arise in different lineages. Each genotype is designated by five amino acids (single amino acid code) arranged in order of sites 73, 120, 132, 136 and 179. Three mutations on the lineage leading from the MCRA (defined using *Geobacter metallireducens* as the outgroup) to *E. coli* are needed to complete the interaction: A120F, V136C and V179I. Genotype FAFCI on the *E. coli* lineage and engineered into *E. coli* IMDH is as active as wild-type. Neither F132V nor C136V affect the performance of *E. coli* IMDH. Simplified from a full tree of 537 taxa.

The two processes can be distinguished by determining the order in which mutations arise during the course of evolution. Phylogenetic analysis suggests that the most recent common ancestor (MRCA) of *E. coli* and *P. aeruginosa* had amino acids FAFVV at sites 73, 120, 132, 136 and 179 (Figure 5). On the lineage leading to *E. coli* mutations V136C and V179I arose first (the order is indeterminate) before mutation A120F. Each mutation is compatible with F73. On the lineage leading to *P. aeruginosa* mutations F73L and F132V arose (the order is indeterminate) before mutation V132L and finally mutation V136I. The presence of V179 is expected to compensate the potentially deleterious interaction between L73 and F132 in the event that the F73L mutation arose first. Hence, the pattern of replacements supports the nearly neutral process because a potentially deleterious mutation never arose before a compensatory mutation in the same lineage.

### Implications of Cryptic Epistasis for Molecular Evolution

Our demonstration of rampant cryptic epistasis in IMDH is entirely in accord with a recent insightful analysis of protein evolution that invoked extensive epistasis to account for the retarded divergence seen in ancient proteins [66]. There the case was made for a rugged fitness landscape characterized by multidimensional sign epistasis that forces sites to be conserved for billions of years until the right combination of amino acids at other sites to allows them to evolve. Our failure to identify compensatory mutations for A94D and A284C is indicative of multidimensional sign epistasis. That a single replacement is sufficient to compensate the F73L mutation demonstrates that epistasis need not always be multidimensional, however.

In an earlier study [67], a mutant library in which 52 natural amino acid replacements from 15 subtilisin orthologues had been recombined was screened for function. Sequence comparisons of the unscreened and the screened libraries suggested that almost all possible pair-wise combinations of amino acids can coexist and that functional co-dependencies are rare. These conclusions seemingly stand in contradiction to ours.

The subtilisin experiment suggests that 7 of 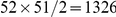 pairs compromise function for 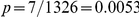. In other words about a half percent of pair-wise interactions are deleterious. For *E. coli* IMDH, the probability, *f*, that introducing an amino acid from an orthologue has no effect on function is 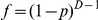, where *D* is the number of residues that differ between the two sequences. For *E. coli* IMDH we have 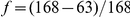, 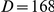 and hence 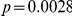. The two-fold difference between the two estimates of *p* is small considering the differences between the enzymes and the experimental methods employed. The take-home lesson is that epistatic interactions may be rare individually, but their cumulative impact on evolution rapidly increases with divergence, *D*. The phenomenon is akin to the snowball effect describing the accumulation of Dobzhanski-Muller incompatibilities during speciation [68].

The simplest model of sequence evolution is a Poisson process in which each site accumulates mutations at a constant rate *λ*. The expected number of mutations accumulated at time *t* is simply *λt* and the variance in the number of substitutions at time *t* is also *λt*. This gives the Poisson molecular clock a characteristic variance to mean ratio of 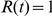. However, sequence analyses show that the molecular clock is the over-dispersed with 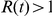 [69]–[75]. Various hypotheses have been proposed to explain this over-dispersion including episodic bursts of selection, increased rates of fixation of deleterious alleles during population bottlenecks, fluctuating neutral spaces and variable mutation rates [73], [76]–[84]. Cryptic epistasis, in causing constraints at sites to vary and hence substitution rates at sites to vary, undoubtedly contributes to over-dispersion in the molecular clock.

Simulations show that ignoring changes in substitution rates (heterotachy) can induce systematic errors in phylogenetic reconstruction, including topological inaccuracies, long-branch biases and other effects [85]–[91]. Simulations also show that ignoring co-dependencies among sites causes the amount of evolution to be underestimated, particularly on branches deep in a tree [92]. The resulting impression of rapid ancient radiations with an indeterminate branching order makes identifying the origins of some taxonomic groups difficult [93], [94]. Taking explicit account of co-dependencies within data has been shown to aid phylogenetic inference [92], [95]. While recent advances accommodate temporal variability in substitution rates within sites [87], even going so far as to model pair-wise interactions between sites in close proximity using predefined statistical potentials calculated from structural data [88], general phylogenetic practice does not [89]. The extensive cryptic epistasis we have revealed suggests that the usual practice of ignoring co-dependencies among sites needs reconsidering.

Ancestral sequence resurrection is a popular experimental approach to explore ancient phenotypes and adaptations [1]. Accurately inferred ancestral sequences are essential, otherwise there can be little confidence in the experimental results. Caution is warranted when interpreting functional patterns that mimic *in silico* reconstruction biases [96]. Current methods ignore functional co-dependencies among sites; the consequences for the accuracy of inferred ancestral sequences is largely unexplored. On the one hand, coupling between sites represents a loss of degrees of freedom (knowing the residue at one site allows inferences to be made about the residues at coupled sites) that leads to overconfidence in reconstructed trees [97]. This is particularly problematic if attempting to reconstruct ancestral sequences during a supposed rapid ancient radiation. On the other hand, the same loss of degrees of freedom means that fewer inferences are made, which should improve accuracy. Simulations suggest that the conditions producing phylogenetic uncertainty also make the ancestral state identical across plausible trees [98]. This helps make ancestral sequence reconstructions robust to phylogenetic uncertainty.

Our dissection of epistatic interactions with site 73 shows that amino acid replacements accumulated during evolution can interact without affecting protein function. Nevertheless, cryptic epistasis may impact functional evolution. Reconstructing ancestral proteins on either side of an ancient functional change neglects epistatic interactions that earlier prevented the change and that later prevented the new function reverting [99] or changing in response to a new selective pressure. Such canalizing epistasis both retards functional evolution and thwarts attempts to engineer enzymes rationally [100]. Protein breeding experiments commonly use mutant libraries, generated either by recombining related sequences [101] or by allowing sequences to accumulate ‘neutral drift’ mutations [102], to circumvent the canalizing effects of cryptic epistasis. We speculate that the rampant cryptic epistasis, inferred by computational methods [66] and detected in experiments on IMDH, might be sufficiently extensive to resist functional changes on evolutionary time scales. Only when rare neutral mutations relieve its canalizing effects can new functions evolve. This model potentially explains why protein evolution is characterized by long periods of functional stasis punctuated by rapid functional shifts.

## Materials and Methods

### Strains, Media, and Chemicals

*E. coli* K12 strains MG1655, JW5807 (Keio Collection) [103], MM294D and BL21-gold-*DleuB::kan^r^* have been previously described [53], [58]. A derivative of *E. coli* strain BL21-gold (Stratagene) was constructed by P1 transduction [104] of the *ΔleuB-leuC::kan^r^* construct from strain JW5807. LB medium was supplemented with 15 g/l Bacto agar for plates [104]. TALON Superflow metal affinity resin and TALON xTractor Buffer were purchased from Takara Bio USA (Madison, WI). Unless specified otherwise, chemicals were purchased from Sigma-Aldrich (St. Louis) and restriction enzymes were purchased from New England Biolabs (Ipswich, MA) and Fermentas (Canada). dl-threo-3-isopropylmalic acid was purchased from Wako Pure Chemical Industries (Japan).

### Sequencing

All mutants sequenced at the BioMedical Genomics Center, University of Minnesota.

### Constructing Plasmid pLeuB

The *leu* operon, from mid *leuA* through *leuC*, was acquired by genomic PCR from MG1655. The genomic PCR product and pMML22KBA-KYVY [58] were digested with restriction enzymes RsrII and SphI. The vector and insert were ligated by quick ligation (Fermentas), to create pLeuB7. The construct was transformed by RbCl transformation [105] into MM294D and selected on LB/Amp(100 µg/ml), overnight at 37°C.

### Primer Design

The 5′-primer is designed with 15–20 bases, then the bases to be mutated, followed by a minimum of 12 bases at the 3′ end. The 3′-primer is complementary to the first 15–20 bases 5′-primer. Thus, the primers are staggered and only the 5′-primer encodes the mutations to be introduced.

### Plasmid Methylation

Cytosine residues in plasmid pLeuB7 were methylated by the CpG Methyltransferase M.SssI (New England Biolabs) according to the manufacture's instructions [106]. Methylated DNA and a nonmethylated control were diluted 1∶25, and 2 µl transformed into *E. coli* strain MM294D by using the RbCl/CaCl_2_ method [105]. After transformation cells were plated on LB/ampicillin (100 µg/ml) and incubated overnight at 37°C.

### Mutagenesis

Restriction sites (Figure S5) and the 168 single mutants were introduced into wild-type *E. coli leuB* using the protocol in Table 2 [107]. Five microliters of each finished reaction was run on a 1% agarose gel to verify the PCR worked, and 2 µl was transformed [105] into MM294D, plated on LB/ampicillin (100 µg/ml) and incubated overnight at 37°C. The presence of mutations was confirmed by sequencing. Mutant enzymes with kinetic characteristics different from wild-type had their entire *leuB* gene re-sequenced to confirm that no other mutations had inadvertently been introduced.

**Table 2:** PCR reaction mixture and cycling protocol.

Reaction mixture		
µl		
2.0	H_2_O	
1.0	10× Hot Start Buffer (Novagen)	
0.6	25 mM MgSO_4_	
1.0	2 mM dNTP's	
4.0	Methylated pLeuB7 template (∼80ng/µl)	
0.6	5′-Primer (0.3 µM)	
0.6	3′-Primer (0.3 µM)	
0.2	KOD Hot Start (Novagen)	
**10.0**		

Cycling protocol repeated 20 times		
95°C	2 mins	Polymerase Activation
95°C	20 secs	Denaturation
60°C	10 secs	Primer Annealing
70°C	3 mins	0.5 min/kb Extension

Double mutants incorporating F73L, A94D and A284C with other mutations were constructed by restriction digestion and ligation using strain MM294D as a host. F73L, A94D, A284C were restriction digested and inserts with L24V, S156E, and Y360A ligated to form parent vectors F73L,L24V, F73L,S156E, A94D,L24V, A94D,S156E, A284C,S156E, and A284C,Y360A. L24V removes the AflII site, S156E removes the BamHI site, and Y360A removes the SnaBI site. Parent vectors were then restriction digested and inserts, obtained from restriction digests of other single mutants, were ligated in. After transformation [105] cells were plated on LB/ampicillin (100 µg/ml) and incubated overnight at 37°C. Colonies were grown in LB/ampicillin (100 µg/ml) and the plasmids purified. Double mutants were identified by the presence of a restored AflII, BamHI or SnaBI restriction site. Those remaining mutations close to F73L, A94D and A284C that could not be introduced by restriction digestion and ligation were introduced by PCR mutagenesis and the entire gene sequenced.

In all 694 mutants were constructed: 17 restriction sites were introduced into pLeuB7, 170 single mutants were made (the exact position of one single amino acid deletion could not be reliably identified and so three mutates deleting residues 150, 151 and 152, were constructed), and 3×169 double mutants were made.

### Protein Expression

Mutant IMDHs were over-expressed from plasmids in a derivative of *E. coli* strain BL21-gold (Stratagene) formed by P1 transduction [104] of the *ΔleuB-leuC::kan^r^* construct from strain JW5807. Transformed cells were grown overnight at 37°C in 5 mL of LB containing ampicillin (100 µg/ml) and 0.2 mM IPTG. Following centrifugation, cells were resuspended in 1 mL of BD TALON xTractor Buffer (Becton-Dickenson). After 10 min rocking at room temperature, the sample was then centrifuged for 20 min at 11,200×*g* and the supernatant transferred to a TALON 2 mL disposable gravity column containing 2 mL of equilibrated BD TALON Superflow metal affinity resin. The protein was then eluted following the manufacture's protocol with the exception that potassium salts were substituted for sodium salts. All enzymes were purified to homogeneity as judged using Coomassie stained SDS-PAGE gels.

### Screening Double Mutants

Double mutants were screened for compensatory mutations at 37°C in 25 mM MOPS, 100 mM KCl, 1 mM DTT, pH 7.3 in the presence of fixed concentrations of 0.2 mM dl-threo-3-isopropylmalic acid and 5 mM MgCl_2_ and 0.1 mM NAD. The concentration of NAD lies far below the *K_m_*s of the single mutants (459±51 mM for F73L, 687±35 mM for A94D, 777±34 mM for A284C). With each mutant unsaturated the rate of the reaction is proportional to *k_cat_/K_m_* making improvements in performance readily detectable.

### Enzyme Kinetics

Kinetics were performed at 37°C in 25 mM MOPS, 100 mM KCl, 1 mM DTT, pH 7.3 in the presence of fixed concentrations of 0.2 mM dl-threo-3-isopropylmalic acid and 5 mM MgCl_2_, and with concentrations of NAD varied from 1/4 to 10× the apparent *K_m_*. Reactions were initiated by adding 10 µL of mutant IMDH (diluted in 50 mM potassium phosphate, 300 mM KCl, 150 mM imidazole, 10 mM β-mercaptoethanol, pH 7.0) to 1 ml of the reaction mix in a 1 cm semi-UV (methylacrylate) cuvette (Fisher Scientific). Reaction rates were determined spectrophotometrically by measuring the production of NADH at 340 nm using a molar extinction coefficient of 6220 M^−1^ cm^−1^, in a thermostated Cary 300 Bio with a 6×6 Peltier block (Varian). Inhibition constants were determined in the presence of varying fixed concentrations of reduced coenzyme. Kinetic parameters *V_max_*, *K_m_* and *V_max_/K_m_* were determined using nonlinear regression as implemented in JMP (SAS Institute). Maximum turnover rates, 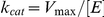, were calculated with enzyme concentrations, [*E*], determined spectrophotometrically by Bradford assay [108] (Bio-Rad) using bovine IgG as the standard.

Each single mutant was independently expressed, purified and kinetically characterized twice.

### Phylogenetics

A total of 537 amino acid sequences (downloaded from GenBank via the NCBI web site http://www.ncbi.nlm.nih.gov/) were aligned using ClustalW software [109]. X-ray structures (IMDHs 1HEX, 1CNZ, 1CM7, IV53, 1W0D, 1WPW, 1VLC, and 1A05) were downloaded from the PDB web site (http://www.pdb.org/pdb/home/home.do) and superimposed using Swiss-Pdb Viewer software [110]. Superpositioned structures were used as a guide to adjust the alignments of highly divergent sequences. A bootstrapped neighbor joining tree was constructed with PHYLIP [111] using a JTT [112] substitution matrix with deep branches swapped and assessed by maximum likelihood. A consensus tree was generated with Mr.Bayes [113] based on a gamma distributed, mixed model of amino acid evolution. The MCMC was run for 75000 generations sampling every 50 generations with a burn-in of 500. Both trees produced similar results when ancestral sites were reconstructed by fastml [114]. With Bayesian posterior probabilities <0.9 accounting for <15% of sites (mostly in flexible loops), the amino acid identities at most sites in the deduced sequence of the most recent common ancestor are reliably inferred.

## Supporting Information

Figure S1 IMDH alignments. *E. coli* and *P. aeruginosa* IMDHs aligned with their most recent common ancestor (MRCA). Single letter amino acid code with dashes for deletions and question marks in the MRCA when Bayesian posterior probabilities fall below 90%. Top numbering refers to the alignment. Bottom numbering refers to *E. coli* IMDH. Asterisks above the alignment denote residues identical in both species. Protein secondary structures, H for helices and S for sheets, are indicated below the alignment.
(0.03 MB DOC)

Figure S2 The evolution of cryptic epistasis. (A) Moving either amino acid from one species into the enzyme of another species risks loss of function when three mutations have occurred, one at each site in one lineage and one at either site in the other lineage because genotypes AB and aB′ are each synthetic combinations. (B) Moving amino acids species B into species A identifies two sites of three engaged in pair-wise interactions (mutants ABC and aBC are synthetic combinations; mutant Abc represents an ancestral functional state).
(0.08 MB TIF)

Figure S3 Mutations affecting kinetic performance are distributed throughout the IMDH structure. Performance scale: red<1%<dark pink<10%<light pink<50%<white<80% of *E. coli* wildtype IMDH. F120A, the only mutation to show increased activity, is shown in green. Asp251 is at the catalytic center of the active site. The homodimer interface, a four helix bundle, is formed with the two helices on the right that have no mutations in them.
(3.86 MB TIF)

Figure S4 The position of F73 in IMDH. The side chain of IMDH residue F73 forms the side of a pocket into which the amide of the nicotiamide ring must bind during catalysis. F73 is replaced by K100, which is essential to catalysis in the related isocitrate dehydrogenase [53]. Coenzyme and substrate modeled into *E. coli* IMDH (pdb 1CM7) [53] from *E. coli* isocitrate dehydrogenase (pdb 1AI2).
(8.14 MB TIF)

Figure S5 Restriction sites introduced into wildtype *E. coli leuB* are silent substitutions. Single cut sites in bold, double cut sites (one cut elsewhere in pLeuB7) in italics.
(0.03 MB DOC)
