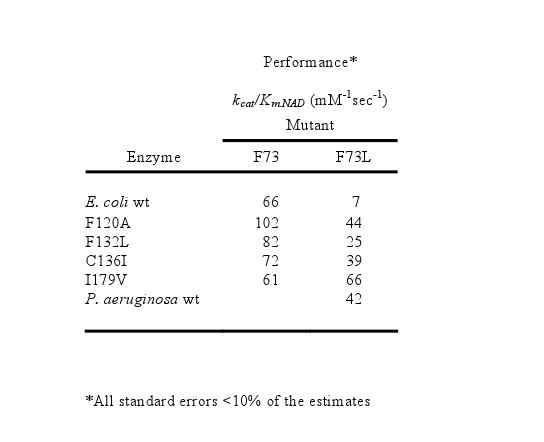# Correction: Pervasive Cryptic Epistasis in Molecular Evolution

**DOI:** 10.1371/annotation/d618ce28-5010-47df-a44e-148ecdc0fef6

**Published:** 2010-11-02

**Authors:** Mark Lunzer, G. Brian Golding, Antony M. Dean

In Table 1, the data in the F73 column are incorrect. See the corrected Table 1 here:

**Figure:**