# Supplementary material for: Pervasive Cryptic Epistasis in Molecular Evolution
Source: PLoS Genet. 2010 Oct 21;6(10):e1001162. doi: 10.1371/journal.pgen.1001162 (PMC2958800; doi:10.1371/journal.pgen.1001162)
Supplement: Figure S1 — IMDH alignments. E. coli and P. aeruginosa IMDHs aligned with their most recent common ancestor (MRCA). Single letter amino acid code with dashes for deletions and question marks in the MRCA when Bayesian posterior probabilities fall below 90%. Top numbering refers to the alignment. Bottom numbering refers to E. coli IMDH. Asterisks above the alignment denote residues identical in both species. Protein secondary structures, H for helices and S for sheets, are indicated below the alignment. (0.03 MB DOC) [file pgen.1001162.s001.doc]

20 40 60

***---*--*********-* --*-***------*------ -----******-**-**---

Pseudo MSK--QILILPGDGIGPEIM AEAVKVLELANDKFQLGFEL AEDVIGGAAIDKHGVPLADE

MRCA MSKTYKIAVLPGDGIGPE?M AEA?KVLDAV??KFGH?FE? ?EA?VGGAAID?HG?PLPEE

Ecoli MSKNYHIAVLPGDGIGPEVM TQALKVLDAVRNRFAMRITT SHYDVGGAAIDNHGQPLPPA

SSSSSSSSSSS HHHHHH HHHHHHHHHHHHHH SSSS SS HHHHHH HH

20 40 60

80 100 120

*-*---******-*-***** *-----*--****-**--*- ---**-*****-**--*---

Pseudo TLERARQADAVLLGAVGGPK WDRIERDIRPERG-LLKIRS QLGLFANLRPAILYPQLADA

MRCA TLE?CK?SDAILFGSVGGPK W??LP?DQRPERGALLPLRK ?FGLF?NLRPAIIFP?LT?A

Ecoli TVEGCEQADAVLFGSVGGPK WEHLPPDQQPERGALLPLRK HFKLFSNLRPAKLYQGLEAF

HHHHHHH SSSSSS HHHHHHHHHHH HH SSSSSSSS

80 100 120

140 160 180

--*-------*-***-**** *********-**-------- -*-**--*---**-****--

Pseudo SSLKPEVVA-GLDILIVREL TGGIYFGQPREQRVLENGER QAYDTLPYSESEIRRIARVG

MRCA SPLK?EIIA?GFDILVVREL TGGIYFGQPKGR?????GE? R?FDTM?Y?VSEIERIARVA

Ecoli CPLRADIAANGFDILCVREL TGGIYFGQPKGR-EGSGQYE KAFDTEVYHRFEIERIARIA

HHHHH SSSSSSSS SSS SSSSSSSSSHHHHHHHHHHH

140 159 179

200 220 240

*--**-*--*--*-****** -**-****-*-*-*--**** **-***-***-***---*-*

Pseudo FDMARVRGKKLCSVDKANVL ASSQLWREVVEEVAKDYPDV ELSHMYVDNAAMQLVRAPKQ

MRCA FEAARKR?KKV?S?DKANVL ?TSVLWREVV?EVAK?YPDV EL?HMYVDNAAMQLV?NPKQ

Ecoli FESARKRRHKVTSIDKANVL QSSILWREIVNEIATEYPDV ELAHMYIDNATMQLIKDPSQ

HHHHHH SSSSSS SSSSSSSSSSSSSSS SSSSSSHHHHHHHHHH

199 219 239

260 280 300

***----*-********--* -***-********-----*- ***--********--****-

Pseudo FDVMVTDNMFGDILSDEASM LTGSIGMLPSASLDANNKGM YEPCHGSAPDIAGQGIANPL

MRCA FDV?LC?NMFGDILSDEAAM LTGSIGMLPSASL?EGGFGL YEP?GGSAPDIAG?GIANPI

Ecoli FDVLLCSNLFGDILSDECAM ITGSMGMLPSASLNEQGFGL YEPAGGSAPDIAGKNIANPI

SSSS HHHHHHHHHHHHH HH SSSSS SS SSS HH

259 279 299

320 340 360

*-***-----***-----** -***-*----*--*-****- ---*---*-*-****-----

Pseudo ATILSVSMMLRYSFSQAAAA DAIEQAVSKVLDQGLRTGDI WSEGCRKVGTQEMGDAVVAA

MRCA AQILSAAMMLRYSF??EEAA DAIEKAVEKVLAQGYRTADI ?????KLVSTKEMGDAIVAA

Ecoli AQILSLALLLRYSLDADDAA SAIERAINRALEEGIRTGDL -ARGAAAVSTDEMGDIIARY

HHHHHHHHHHHHHH HHHH HHHHHHHHHHHH HHHHHHHHHHH

319 339 358

365

-----

Pseudo LKNL-

MRCA L????

Ecoli VAEGV

HH

363
