# Supplementary figures and images for: Pervasive Cryptic Epistasis in Molecular Evolution
Source: PLoS Genet. 2010 Oct 21;6(10):e1001162. doi: 10.1371/journal.pgen.1001162 (PMC2958800; doi:10.1371/journal.pgen.1001162)

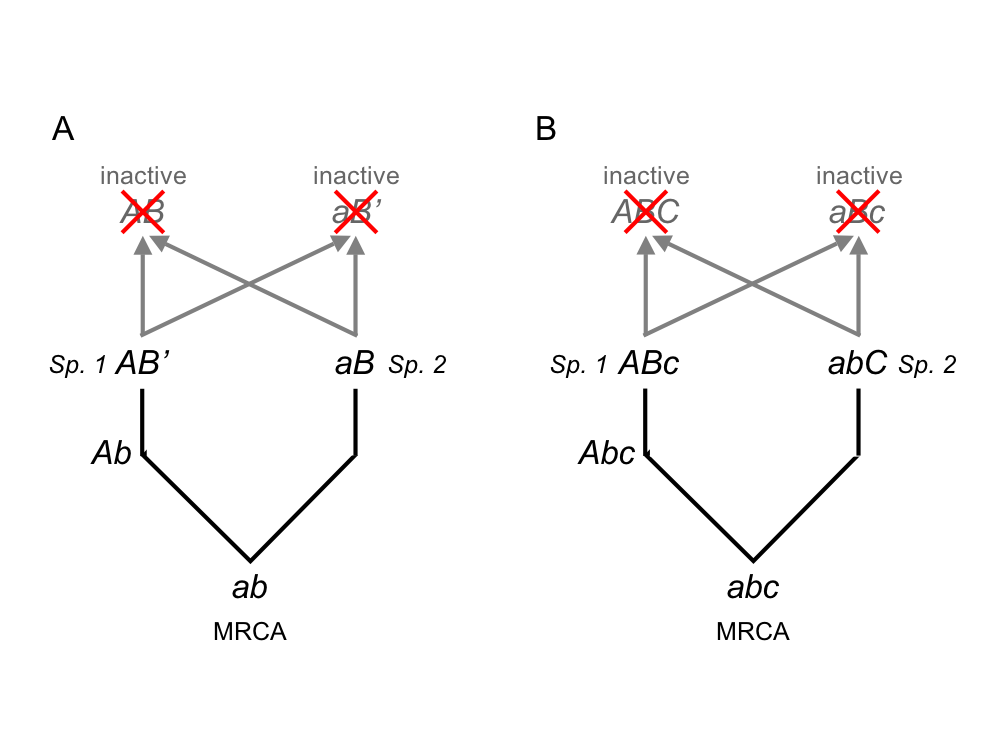

Supplement: Figure S2 — The evolution of cryptic epistasis. (A) Moving either amino acid from one species into the enzyme of another species risks loss of function when three mutations have occurred, one at each site in one lineage and one at either site in the other lineage because genotypes AB and aB′ are each synthetic combinations. (B) Moving amino acids species B into species A identifies two sites of three engaged in pair-wise interactions (mutants ABC and aBC are synthetic combinations; mutant Abc represents an ancestral functional state). (0.08 MB TIF) [file pgen.1001162.s002.tif]

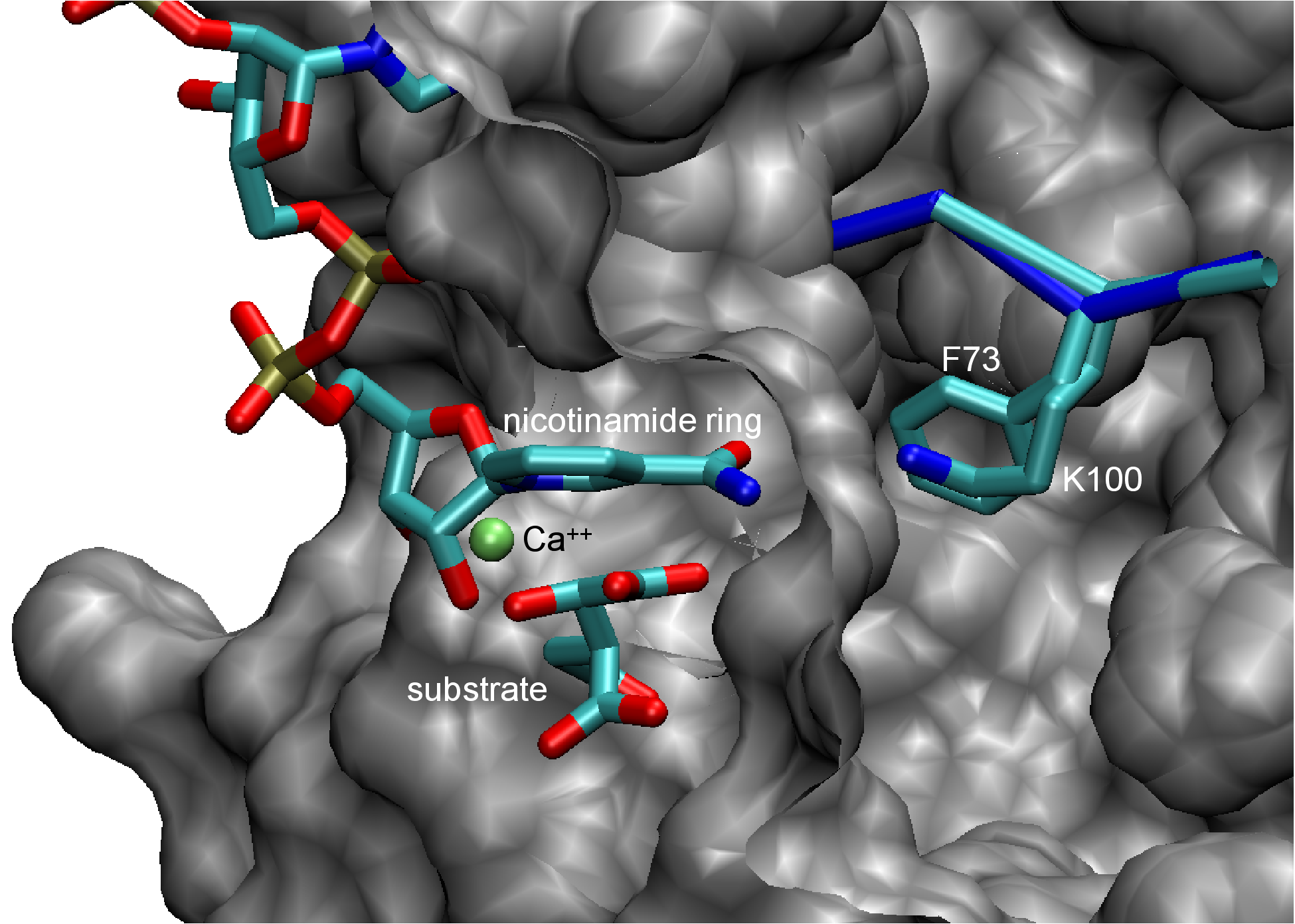

Supplement: Figure S4 — The position of F73 in IMDH. The side chain of IMDH residue F73 forms the side of a pocket into which the amide of the nicotiamide ring must bind during catalysis. F73 is replaced by K100, which is essential to catalysis in the related isocitrate dehydrogenase [53]. Coenzyme and substrate modeled into E. coli IMDH (pdb 1CM7) [53] from E. coli isocitrate dehydrogenase (pdb 1AI2). (8.14 MB TIF) [file pgen.1001162.s004.tif]
