# Supplementary material for: Pervasive Cryptic Epistasis in Molecular Evolution
Source: PLoS Genet. 2010 Oct 21;6(10):e1001162. doi: 10.1371/journal.pgen.1001162 (PMC2958800; doi:10.1371/journal.pgen.1001162)
Supplement: Figure S5 — Restriction sites introduced into wildtype E. coli leuB are silent substitutions. Single cut sites in bold, double cut sites (one cut elsewhere in pLeuB7) in italics. (0.03 MB DOC) [file pgen.1001162.s005.doc]

*XmaI*

1 ATGTCGAAGA ATTACCATAT TGCCGTATTG *CCCGGG*GACG GTATTGGTCC GGAAGTGATG ACCCAGGCGB

M S K N Y H I A V L P G D G I G P E V M T Q A L

**AflII** **FspAI**

71 **TTAAG**GTGCT GGATGCCGTG CGCAACCGCT TTGCG**ATGCG CAT**CACCACC AGCCATTACG ATGTAGGCGG

K V L D A V R N R F A M R I T T S H Y D V G G

141 CGCAGCCATT GATAACCACG GGCAACCACT GCCGCCTGCG ACGGTTGAAG GTTGTGAGCA AGCCGATGCC

A A I D N H G Q P L P P A T V E G C E Q A D A

*ApaI*

211 GTGCTGTTTG GCTCGGTAGG C*GGGCCC*AAG TGGGAACATT TACCACCAGA CCAGCAACCA GAACGCGGCG

V L F G S V G G P K W E H L P P D Q Q P E R G A

**HindIII**

281 CGCTGCTGCC TCTGCGTAAG CACTTC**AAGC TT**TTCAGCAA CCTGCGCCCG GCAAAACTGT ATCAGGGG*CT*

L L P L R K H F K L F S N L R P A K L Y Q G L

*XhoI*  *SacI*

351 *CGAG*GCATTC TGTCCGCTGC GTGCAGACAT TGCCGCAAAC GGCTTCGACA TCCTGTGTGT GCGC*GAGCT****C***

E A F C P L R A D I A A N G F D I L C V R E L

**SgrAI**  **BamHI**

421 **ACCGGCG**GCA TCTATTTCGG TCAGCCAAAA GGCCGCGAA**G GATCC**GGACA ATATGAAAAA GCCTTTGATA

T G G I Y F G Q P K G R E G S G Q Y E K A F D T

**BstZ17I**

**AccI**  **BstBI**  **AfeI**

491 CCGAG**GTATA C**CACCGT**TTC GAA**ATCGAAC GTATCGCCCG CATCGCGTTT GAA**AGCGCT**C GCAAGCGTCG

E V Y H R F E I E R I A R I A F E S A R K R R

**ZraI** **HpaI**

**AatII** *PstI*  **HincII**

561 CCACAAAGTG ACGTCGATCG ATAAAGCCAA CGTG*CTGCAG* TCCTCTATTT TATGGCGGGA GATC**GTTAAC**

H K V T S I D K A N V L Q S S I L W R E I V N

**NdeI BsrGI**  **MfeI**

631 GAGATCGCCA CGGAATACCC GGATGTCGAA CTGGCG**CATA TGTACA**TCGA CAACGCCACC ATG**CAATTG**A

E I A T E Y P D V E L A H M Y I D N A T M Q L I

701 TTAAAGATCC ATCACAGTTT GACGTTCTGC TGTGCTCCAA CCTGTTTGGC GACATTCTGT CTGACGAGTG

K D P S Q F D V L L C S N L F G D I L S D E C

**NheI**

**BmtI**

771 CGCAATGATC ACTGGCTCGA TGGGGATGTT GCCTTCC**GCT AGC**CTGAACG AGCAAGGTTT TGGACTGTAT

A M I T G S M G M L P S A S L N E Q G F G L Y

*NgoMIV*

841 GAACCG*GCCG GC*GGCTCGGC ACCAGATATC GCAGGCAAAA ACATCGCCAA CCCGATTGCA CAAATCCTTT

E P A G G S A P D I A G K N I A N P I A Q I L S

911 CGCTGGCACT GCTGCTGCGT TACAGCCTGG ATGCCGATGA TGCGGCTTGC GCCATTGAAC GCGCCATTAA

L A L L L R Y S L D A D D A A C A I E R A I N

**SfoI**

**NarI**

**KasI**

**BsiWI**  **BbeI**

981 CCGCGCATTA GAAGAAGGCA TT**CGTACG**GG GGATTTAGCC CGT**GGCGCC**G CCGCCGTTAG TACCGATGAA

R A L E E G I R T G D L A R G A A A V S T D E

**SnaBI**

1051 ATGGGCGATA TCATTGCCCG C**TACGTA**GCA GAAGGGGTGC ATCATCATCA TCATCATTAA TCATGGCTAA

M G D I I A R Y V A E G V H H H H H H *
